# Infectious Diseases

**Published:** 1892-06

**Authors:** R. A. Chudleigh


### INFECTIOUS DISEASES.

Sir Thomas Watson thus writes upon this subject:

"The contagion of scarlet fever lurks about an apartment, or clings to furniture and clothes, for a very long time, even after some care has been taken to purify them. Of this I have known several remarkable examples. You (my pupils) will be asked at what period the danger of imparting the disease is over; I would recommend you to answer that you do not know. I am sure I do not; and therefore I always decline the responsibility of giving an opinion on the matter." In spite of this counsel of caution from the late Queen's physician, I will venture to state my conviction that the virus of scarlet fever may escape disinfection, and may be carried 150 miles in a letter, not merely a day, but even a year after the date of its generation. In virulence, subtlety, and tenacity of life, the virus of scarlet fever seems second only to that of small-pox. The general rule is that infection ceases in seven weeks, or to be quite safe, eight weeks, after the appearance of the rash, provided always that the throat be healed and the skin peeled for so long as those two symptoms linger on there is danger of infection. To this general rule there are, however, decided exceptions, so amply attested that it is prudent to accept them. If there be any fear of infection from the letter mentioned in the query, I would advise those who have been exposed to the contagion to take three drops of belladonna in water, night and morning for three days, and, after missing three days do it again. The homœopathic tincture of belladonna is the most convenient preparation.—R. A. CHUDLEIGH, M. D.

